# Brain Functional Networks in Type 2 Diabetes Mellitus Patients: A Resting-State Functional MRI Study

**DOI:** 10.3389/fnins.2019.00239

**Published:** 2019-03-19

**Authors:** Jian Xu, Fuqin Chen, Taiyuan Liu, Ting Wang, Junran Zhang, Huijuan Yuan, Meiyun Wang

**Affiliations:** ^1^Department of Medical Information Engineering, School of Electrical Engineering and Information, Sichuan University, Chengdu, China; ^2^School of Information Engineering, Hubei University for Nationalities, Enshi, China; ^3^Department of Medical Imaging, Henan Provincial People’s Hospital, Zhengzhou, China; ^4^Department of Computer Science, Chengdu University of Information Technology, Chengdu, China; ^5^Department of Endocrinology, Henan Provincial People’s Hospital, Zhengzhou, China

**Keywords:** type 2 diabetes mellitus, resting-state functional magnetic resonance imaging, graph theory, functional network, small-world, topological properties

## Abstract

**Background:**

Previous diabetes mellitus studies of cognitive impairments in the early stages have focused on changes in brain structure and function, and more recently the focus has shifted to the relationships between encephalic regions and diversification of network topology. However, studies examining network topology in diabetic brain function are still limited.

**Methods:**

The study included 102 subjects; 55 type 2 diabetes mellitus (T2DM) patients plus 47 healthy controls. All subjects were examined by resting-state functional magnetic resonance imaging (rs-fMRI) scan. According to Automated Anatomical Labeling, the brain was divided into 90 anatomical regions, and every region corresponds to a brain network analysis node. The whole brain functional network was constructed by thresholding the correlation matrices of the 90 brain regions, and the topological properties of the network were computed based on graph theory. Then, the topological properties of the network were compared between different groups by using a non-parametric test. Finally, the associations between differences in topological properties and the clinical indicators were analyzed.

**Results:**

The brain functional networks of both T2DM patients and healthy controls were found to possess small-world characteristics, i.e., normalized clustering coefficient (γ) > 1, and normalized characteristic path length (λ) close to 1. No significant differences were found in the small-world characteristics (σ). Second, the T2DM patient group displayed significant differences in node properties in certain brain regions. Correlative analytic results showed that the node degree of the right inferior temporal gyrus (ITG) and the node efficiencies of the right ITG and superior temporal gyrus of T2DM patients were positively correlated with body mass index.

**Conclusion:**

The brain network of T2DM patients has the same small-world characteristics as normal people, but the normalized clustering coefficient is higher and the normalized characteristic path length is lower than that of the normal control group, indicating that the brain function network of the T2DM patients has changed. The changes of node properties were mostly concentrated in frontal lobe, temporal lobe and posterior cingulate gyrus. The abnormal changes in these indices in T2DM patients might be explained as a compensatory behavior to reduce cognitive impairments, which is achieved by mobilizing additional neural resources, such as the excessive activation of the network and the efficient networking of multiple brain regions.

## Introduction

Diabetes is a metabolic disease characterized by long-term hyperglycemia, which may cause various complications such as microvascular disease, retinopathy, kidney disease and peripheral neuropathy. In severe cases, diabetes can be fatal. Statistics produced by the International Diabetes Federation in 2015 indicated that the number of diabetes patients was about 415 million, and predicted that it would reach up to 642 million by 2040 (Ogurtsova et al., 2017). In China, the rate of diabetes cases has approximately doubled in the last 10 years. The incidence of diabetes in China is 11.6% and the number of diabetics is more than 100 million, both of which are the highest in the world. Type 2 diabetes mellitus (T2DM) patients account for around 90% of cases of diabetes mellitus (Holman et al., 2015), the morbidity of which has been increasing over the years. Consequently, diabetes has become one of the most critical health issues in China (Ning, 2018).

Previous studies have shown that T2DM can result in various cognitive deficits in the early stages of the disease, including lapses in concentration, hypomnesis, visual impairment, and declines in information processing speed and executive capacity (McCrimmon et al., 2012). In extreme cases, these cognitive deficits can develop into dementia. At present, the rapidly rising incidence of diabetes and its associated cognitive impairments has become a major issue. Although some progress has been made with regard to determining the cognitive impairments caused by T2DM, the underlying neuronal mechanisms of the disorder are still not well understood.

Recently, resting-state functional magnetic resonance imaging (rs-fMRI) has been used to study the underlying pathogenesis of many kinds of central nervous system diseases, including those which affect brain metabolism (Zhang L.J. et al., 2014; Zhou X.Q. et al., 2014). As an emerging non-invasive diagnostic tool, rs-fMRI can be applied to explore and distinguish impaired and normal cognitive function in patients with diabetes mellitus.

The brain network analysis method based on graph theory is mainly used to explore the potential mechanism of normal human brain and various brain diseases, and it is found that the brain function networks of normal people and brain diseases patients have small-world characteristics (Supekar et al., 2009; Zhang et al., 2011; Lei et al., 2015). He et al. (2007) successfully built the first human brain structure network in 2007 and found that it has small-world characteristics. They found that the brain network topology of AD patients has changed (He et al., 2008). They also confirmed the existence of stable small-world characteristics by studying the brain structure network of multiple sclerosis patients (He et al., 2009). Since then, this method has been adopted in many researches (Zhang et al., 2011; Suo et al., 2015; Xiao et al., 2015).

The graph theory-based network analysis has only been used in patients with T2DM to investigate brain structure. Zhang L.J. et al. (2014) discovered that the white matter network topology (including the efficient of global properties and central sulcus on right side cover) of T2DM patients is changed, and this kind of abnormal network structure is associated with the impairment of executive function observed in these patients. By taking advantage of fiber tracer diffusion magnetic resonance imaging and the method of graph theory, Reijmer et al. (2013) and others found that both local and global network properties of T2DM patients are changed. The abnormal network structure was associated with the information processing speed of patients and was independent of age, sex, education, white matter hyperintensities, lacunar infarct and other factors. There have not been any reports of the changes of brain gray matter functional network topology properties in T2DM patients. Cui et al. (2015) analyzed the differences in default mode network (DMN) of T2DM patients, finding out that T2DM patients were associated with impaired DMN function. With independent component analysis (ICA) methods, Chen et al. (2015) found abnormal functional connections between DMN, left frontal network and sensorimotor network in T2DM patients, but found no abnormal functional connections in other resting-state networks. With the same methods, Xia et al. (2015) investigated whether the attention network had changed in T2DM patients, and explored the relationship between abnormal functional connection of attention network and cognitive behavior.

The rs-fMRI brain network analysis method based on graph theory has become one of the hotspots in the study of normal human brain and neuropsychiatric diseases. It can explore the functional connections between the whole brain and the local brain regions, but it has not been widely used in the study of diabetes. Understanding the abnormal patterns of the brain’s functional network can help to find a new way to treat and evaluate the diagnosis of diabetes.

In the present research, we compared the diversity of entire brain functional network topology properties between T2DM patients and healthy controls by adopting graph theory-based brain network analysis together with rs-fMRI. We also explored the relationship between the changes in brain functional network topology properties and clinical variables and the neurocognitive scale, and discussed the influence of relative cognitive obstacles and possible mechanism that it may have on patients. The results of the present study will provide a theoretical basis for diabetic pathophysiology and clinical presentation, and evidence for the need for early treatment and prognostic evaluations.

## Materials and Methods

### Subjects

The study subjects were selected from a total of 154 potential participants: 91 patients with T2DM at Henan Provincial People’s Hospital, and 63 healthy volunteers from the physical examination center. Fifty two subjects were excluded from the study, including 36 patients with T2DM (5 patients with incomplete clinical data, 6 patients with excessive head movement during fMRI scan and 25 patients who did not meet the inclusion criteria), and 16 healthy controls (1 individual with excessive head movement (translational movement > 1.5 mm or rotation > 1.5) during fMRI scan and 15 subjects that failed to meet the inclusion criteria). The remaining 102 subjects were included in the study: 55 patients in the T2DM group and 47 in the healthy control group. All subjects were right-handed, and were informed of the specific content of the study and voluntarily signed informed consent.

The inclusion criteria are as follows: (1) after oral glucose tolerance test (OGTT) T2DM was diagnosed in accordance with the diagnostic criteria published by the World Health Organization in 1999 (American Diabetes Association, 2014); (2) age 40–75, course of T2DM > 1 year. The exclusion criteria were as follows: (1) functional insufficiency of heart, lung, liver and kidney; (2) hyperthyroidism, hypothyroidism and other systemic diseases; (3) infection, ketoacidosis, hyperosmolarity, severe hypoglycemic coma or other urgent complication; (4) anxiety, depression or other neuropsychiatric disease that affects cognitive function; (5) cerebral hemorrhage, cerebral infarction, cerebral trauma, vascular dementia or other disease or medical history that incurs central nervous system injury; (6) recently taken medication that is likely to affect cognitive function; (7) drug taking or alcohol dependence; (8) failing to complete required test items because of intolerance or other factors; (9) MRI contraindication.

The inclusion criteria of healthy controls (Healthy Controls Corresponding to Type 2 Diabetes Mellitus, T2HC) were as follows: (1) age, gender, highest level of education and handedness are matched to those of the T2DM patients; (2) the OGTT result does not conform to the diagnostic criteria of T2DM published by the World Health Organization in 1999; (3) Montreal Cognitive Assessment Scale (MoCA) grade is normal. The exclusion criteria are the same as described above.

### Clinical and Cognitive Scale Test

Before fMRI scanning, the following clinical characteristics were recorded: gender, age at diagnosis, diabetic course, MoCA, height, weight, glycosylated hemoglobin (HbAlc), fasting blood glucose, total cholesterol (TCHOL), glycerin trilaurate (TG), high density lipoprotein (HDL), and low density lipoprotein (LDL). The course of T2DM was defined as the time elapsed from when the patient was diagnosed with T2DM to the time of fMRI scanning. Height and weight were used to calculate body mass index (BMI) of subjects: BMI = weight (kg)/height (m^2^). BMI < 18.5 is defined as slim, BMI = 18.5–23.9 is normal, BMI ≥ 24 is overweight, BMI = 24–26.9 is fat, BMI = 27–29.9 is corpulent, BMI ≥ 30 is severely obese, BMI ≥ 40 is extremely obese.

The MoCA was used to evaluate the integral cognitive function of all subjects, and the test was performed according to standard procedures. The test was carried out in a quiet environment. At the same time, subjects were expected to be relaxed, conscious and non-contradictory. The test assessed 8 cognitive domains: visual space and executive function, attention, memory, naming, abstract thinking, language, delayed recall and orientation. The MoCA is commonly used to screen for Mild Cognitive Impairment (MCI), for which it displays high sensitivity (Hobson, 2015). The MoCA test result has a total score of 30 points, with a final score ≥ 26 being considered normal.

### Rs-fMRI Data Acquisition

Subjects were scanned under resting conditions using a 3.0T superconducting magnetic resonance imaging system and a Siemens 8-channel head coil. During scanning, subjects were instructed to take the supine position, close their eyes but do not sleep, and try to keep their body motionless. Head movement was limited with a foam pad, and the subject’s hearing was protected with foam earplugs or earphones. First, regular MRI scanning was conducted to detect brain abnormalities, then echo planar imaging (EPI) was used to collect resting-state brain function. The scan parameters were as follows: TR = 2000 ms, TE = 30 ms, seam thickness = 5 mm, FOV = 240 × 240 mm^2^, matrix = 64 × 64, voxel size = 3.75 × 3.75 × 5 mm^3^, FA = 90°, acquisition 210 time points, the total scan time is 420 s. Subjects were asked to close their eyes, relax and keep their head motionless, and try to keep a clear head without much thinking before the functional scanning. After the scan was complete, the respondents were asked to cooperate, and those who did not cooperate well were excluded from the study.

### Data Preprocessing

Data preprocessing analysis was conducted in the MATLAB 2017a software environment by using the GRETNA graph-based network analysis toolkit. First, data from the first 10 time points were removed to exclude the problem of magnetic field uniformity, then time layer correction of rs-fMRI data was performed, and moving 3 mm horizontally or rotating in the direction of x, y, and z axis as standard strip head dynamic data. The fMRI images were normalized after head-movement correction by using the MNI-152 standard template of brain anatomy of the Neurological Research Institute, Montreal, Canada, by matching the structure and function images of the subjects and resampling data (voxel size was 3 mm × 3 mm × 3 mm). Next, linear drift and low frequency filter (frequency 0.01–0.08 Hz) were taken to correct the influence of linear frequency drift and high-frequency physical noise; doing Gaussian smoothing to normalized graph, it was treated with 4 mm height and a half full width, smooth kernel size is 4 × 4 × 4. Finally, removal covariate included the impact of 6 head parameters (3 translation parameters and 3 rotation parameters), the whole mean signal, white matter, cerebrospinal fluid signal, age and gender.

### Network Construction

#### Node Definition

A network is composed of many nodes and the edges of the connection between these nodes. In brain networks, nodes represent brain regions and edges represent the degree of statistical dependence of blood oxygenation level dependent (BOLD) imaging between different brain regions. In the article, brain is divided into 90 cortices and cortical interest areas.

#### Edge Definition

For every subject, we chose average time list of 90 brain regions, then, calculated the correlation coefficient between the mean time series value of the two brain regions (spread all brain regions, such as the i-th brain area and the j-th brain area), which as the functional connection metric between them. Last, obtained was a 90 × 90 correlation matrix, which is called a weighed functional connection matrix. According to the predefined threshold (see threshold selection below), we converted the weighed functional connection matrix to a binary adjacency matrix. If the absolute value of the correlation coefficient between any two brain regions was less than the given threshold, it was recorded as 0, otherwise it was recorded as 1.

### Network Analysis

#### Threshold Selection

The number of sides of each brain network is different. To correct for this difference, we applied the sparse threshold (S) range to the relative matrix to ensure the brain network of every subject contained the same number of sides. For every subject, S is defined as the ratio between the actual number of edges and the highest possible number of edges. Because there has not any exact means to define the choice of single threshold at present, previous brain network analysis study used the range of S to thresholding every relative matrix repeatedly. The selection criteria should meet the following two conditions: (1) the minimum of S should meet average node degree in every thresholding network that id 2log(N), and N is node number; (2) the maximum of node should be satisfied with small-world characteristics scalar σ and that is bigger than 1.1. After above process, it would produce a range of S, that is pitch is 0.01 and S between 0.1 and 0.34. Network of this threshold range produced can guarantee small-world characteristics estimation with sparse attribute and minimal pseudo-side. The subsequent brain network analysis will calculate the global network properties and node properties in the order of each sparsity level.

#### Network Parameters

Global properties include: (1) small-world parameters, which includes cluster coefficient, characteristic path length, normalized cluster coefficient, normalized characteristic path length, and small-world characteristics; (2) network efficiency, which includes global efficiency, and component efficiency. Node properties include: (1) node degree; (2) node efficiency; and (3) the betweenness of node.

### Statistical Analysis

#### Demographic and Clinical Data Statistical Analysis

Statistical analysis of the clinical data of the subjects included in the study was performed by using the SPSS 22.0 software package. The chi-squared test was used to compare the gender differences between groups. The measurements of other variables (age, BMI, course of disease, glycosylated hemoglobin, fasting blood glucose, total cholesterol, triglyceride, high density lipoprotein, low density lipoprotein and MoCA analog scale) were analyzed by using two-sample *t*-tests. *p* < 0.05 was deemed to be statistically significant.

#### Statistical Test of Network Properties Between Groups

**Figure 1:**
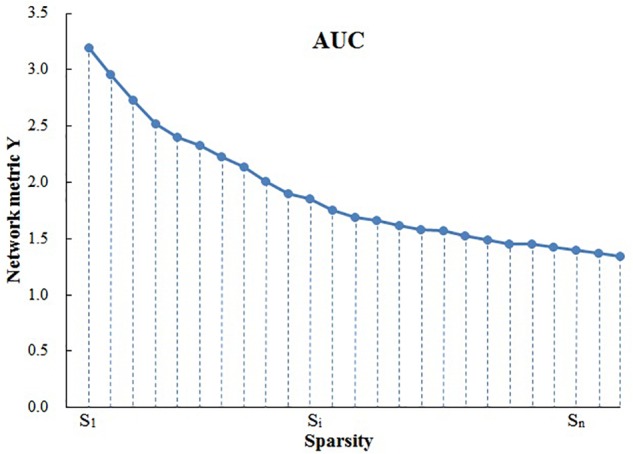
The calculation of area under the curve (AUC). S_1_ = 0.10, S_*n*_ = 0.34, Δ*S* = 0.01.

Because area under the curve (AUC) describes the topology properties of brain network generally, and it can select single threshold calculation independently, moreover, it is highly sensitive about topology structure of brain disease abnormally. As a result, AUC of every topology attribute was selected as statistical sample (Suo et al., 2015). The AUC for a general metric Y can be defined as the AUC for calculating the sparsity range from S1 to Sn (the interval is Δ*S*) (Figure 1), and it’s formula can be described as Lei et al. (2015):

$$Y^{\text{AUC}}\;=\;\frac{1}{2}\;\sum_{k=1}^{N-1}\;[Y(S_{k})\;+\;Y(S_{k-1})]\;•\;\Delta S$$

To determine whether there are inter-group differences in network properties, the non-parametric permutation test is used to test the AUC of each network attribute in the two sets of samples. It is divided into the following steps:

(1) Building the null hypothesis. It is assumed that there is no difference between the means of the two statistical samples.
(2) Determining inspection level α, which is designated as 0.05.
(3) Calculating test statistic of previous two groups’ samples.
(4) To determine whether the differences between groups of network properties could occur accidentally, a sample is selected at random without replacement from sample observation (two samples get together) so as to regroup, then replacement statistical tests of two samples, AUC after stochastic grouping were computed.
(5) Setting the number of random packets, for example 10,000 times, repeat step 4 1,000 times, then get a empirical exampling distribution of replacement statistical tests.
(6) Adopting 95% of every empirical exampling distribution as critical value of null tail test of the null hypothesis. This kind of mistakes is kept within 0.05, and calculating odds is *p*.
(7) With regard to the inspection level (significance level) given by step 2, and according to the principle of small probability, conclusion is drawn.

It is worthwhile to note that before taking statistical test, the effects of age, sex and other parameters need to be removed by multiple linear regression to ensure the differences between groups of every network properties are caused by diseases. To solve multiple comparative problems, use calibration methods of false discovery rate (FDR) proposed by Benjamini-Hochberg, which is to correct the *p*-value after difference between each network attribute group.

#### Correlation Analysis Between Network Properties and Clinical Parameters

After determining the differences in network properties between the groups, we examined the relationship between these differences and the clinical parameters of T2DM, with the age and gender as cointegration variables. Clinical parameters include BMI, course of disease, glycated hemoglobin, fasting blood-glucose, total cholesterol, triglyceride, high-density lipoprotein, low-density lipoprotein, and MoCA score.

## Results

### Demographic Data, Clinical Data, and Cognitive Scale

The results of demographic data, clinical features and cognitive scales of all subjects are shown in Table 1. The MoCA grade of the T2DM group is greater than 26, which is seen as cognitive dysfunction. There were no significant differences between the T2DM and T2HC groups in gender, age, BMI, TCHOL, TG, or LDL, but there were differences between the groups in HDL and MoCA scores.

**Table 1:** Demographic data and clinical Features of type 2 diabetes mellitus patients and the corresponding control group.

Characteristics	Mean ±*SD*		*P*-value
	Type 2 diabetes mellitus (*n* = 55)	Control group (*n* = 47)	
Gender (male/female)	35/20	21/26	0.073
Age	53.31 (± 9.05)	53.34 (± 7.68)	0.919
Course of disease (years)	8.87 (± 6.42)	–	0.112
BMI	25.37 (± 2.85)	25.20 (± 2.80)	0.761
HbA1c	8.15 (± 1.78)	–	**0.001**
GLU	9.59 (± 3.04)	–	0.842
CHOL	4.42 (± 1.10)	4.81 (± 0.87)	0.054
TG	2.05 (± 2.41)	1.55 (± 0.82)	0.152
HDL	0.98 (± 0.24)	1.21 (± 0.33)	**0.001**
LDL	2.51 (± 0.90)	2.77 (± 0.64)	0.100
MoCA	25.36 (± 1.74)	27.79 (± 1.90)	**0.001**

*All calculations were carried out in SPSS 22.0. Double-tailed chi-squared test was used for gender variables. Other measurement variables were tested with two-sample t-test. For all tests, p < 0.05 was considered statistically significant. Values in bold indicate statistically significant differences. BMI, body mass index; HbA1c, glycated hemoglobin; GLU, fasting blood glucose; CHOL, cholesterol; TG, triglyceride; HDL, high-density lipoprotein; LDL, low-density lipoprotein; MoCA, Montreal Cognitive Assessment score.*

### Small-World Brain Functional Network

**Figure 2:**
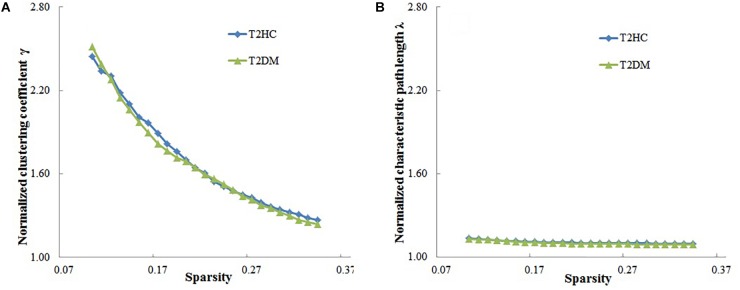
Relationship between the small-world characteristics parameters of brain networks (normalized cluster coefficient and normalized characteristic path length) and sparsity of the brain function network. **(A)** Normalized cluster coefficient (γ) is > 1, and **(B)** normalized characteristic path length is close to 1, which shows that the brain functional networks in both groups have small-world characteristics. T2DM, type 2 diabetes mellitus patients; T2HC, matched healthy controls.

In the given threshold range, compared with the random network, the brain function networks of the two groups have small-world characteristics, i.e., the normalized cluster coefficient is >1, and the normalized feature path length is close to 1 (Figure 2). These results are consistent with previous studies of small-world networks.

### Comparison of Network Topology Properties Between T2DM Patients Group and Healthy Controls

**Figure 3:**
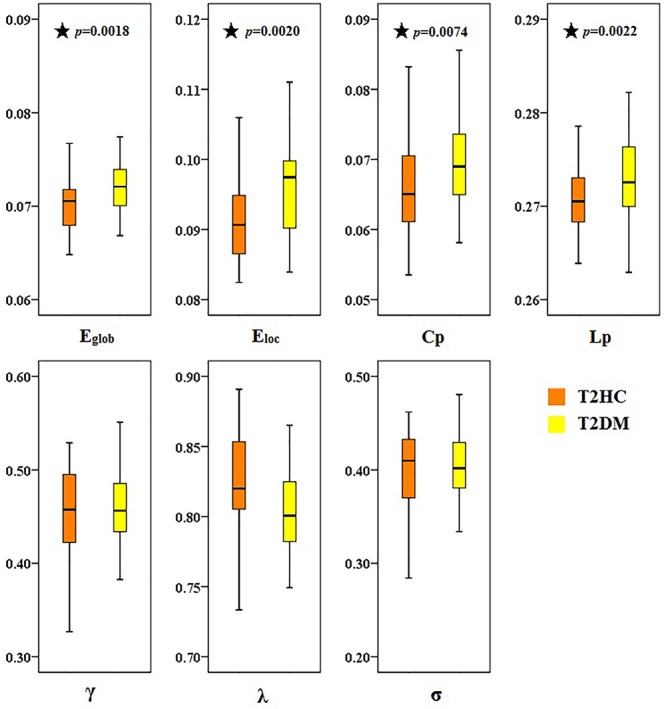
Topological differences in brain function networks between type 2 diabetes mellitus (T2DM) and matched healthy control (T2HC) groups. Compared with the T2HC group, the global properties (E_glob_), local attribute (E_loc_), and cluster coefficient (C_p_) of the T2DM group were higher, and the characteristic path length (L_p_) was lower. There were no significant differences between groups in normalized cluster coefficient (γ), normalized characteristic path length (λ), or small-world characteristics (σ). The ordinate is the area under the curve (AUC) value for each attribute. Black asterisks indicate a significant difference between the two groups.

There was at least one brain region with significant difference in the comparison between record node properties groups (after correlation of FDR, *p* < 0.05). Compared with T2HC, T2DM patients displayed higher global properties (E_glob_), local attribute (E_loc_) and cluster coefficient (C_p_), and lower characteristic path length L_p_ (Figure 3). There was no significant difference between the groups in normalized cluster coefficient (γ), normalized characteristic path length (λ) or small-world characteristics (σ) (*p* > 0.05).

Compared with those of the T2HC control group, the T2DM group displayed increased node degree in the following brain regions: bilateral lenticular putamen and right inferior temporal gyrus (ITG). The T2DM group displayed increased node efficiency in the following brain regions: left central sulcus, left insula, bilateral lenticular putamen and right ITG. The T2DM group displayed increased betweenness of node in the following brain region: right ITG (Table 2).

**Table 2:** Brain areas with significant differences in node centricity between the Type 2 diabetes mellitus (T2DM) and matched healthy control (T2HC) groups.

Brain region	*P*-value		
	Node betweenness	Node degree	Node efficiency
**T2HC < T2DM**			
Left rolandic operculum	0.3123	0.0110	**0.0026**
Left insula	0.3203	0.0072	**0.0022**
Left putamen	0.4287	**0.0014**	**0.0004**
Right putamen	0.3673	**0.0002**	**0.0002**
Right inferior temporal gyrus	**0.0020**	**0.0016**	**0.0008**

*Values in bold indicate statistically significant differences (correlation of FDR, expected threshold level α = 0.05). T2DM, type 2 diabetes mellitus; T2HC, matched healthy controls.*

### Correlation Analysis Between Abnormal Network Topology Properties and Clinical Parameters

**Figure 4:**
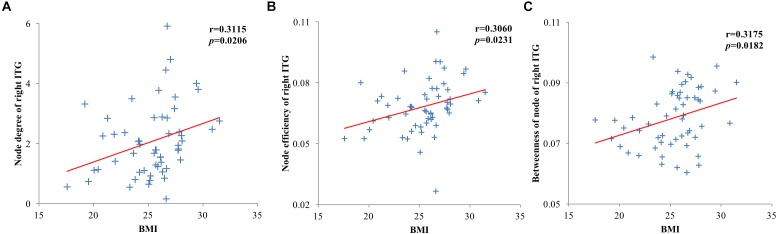
Scatter plots of brain region and clinical index node properties with statistically significant correlations. **(A)** Node degree of the right inferior temporal gyrus of the type 2 diabetes mellitus (T2DM) group was positively correlated with body mass index (BMI); **(B)** Node efficiency of the right inferior temporal gyrus of the T2DM group was positively correlated with BMI; **(C)** The betweenness of node of the right inferior temporal gyrus of the T2DM group was positively correlated with BMI.

The node degree, node efficiencies and the betweenness of node of the right ITG of the T2DM group were positively correlated with BMI (*r* = 0.3115, *p* = 0.0206; *r* = 0.3060, *p* = 0.0231; *r* = 0.3175, *p* = 0.0182, Figure 4). There was no significant correlation between all global and other node properties and all clinical indicators (*p* > 0.05).

## Discussion

This study examined the topological differences in brain function networks of T2DM patients, based on graph theoretical analysis of rs-fMRI data. The main results were as follows: (1) Global properties: the brain function network of T2DM has small-world characteristics as healthy controls. (2) Node properties: compared with healthy controls, the T2DM group have notable changes in insula, lenticular putamen, central sulcus, ITG and other brain regions. (3) Related analysis: the node degree of the right ITG, and the node efficiency of the right inferior and superior temporal gyrus of the T2DM group are all positively correlated with BMI. The excessive activation indices of the T2DM group may be explained as a compensatory behavior, which may reduce cognitive impairment by mobilizing additional neural resources. These findings provide a new perspective of how changes in brain functional topology properties may be related to cognitive function.

### Change of Global Properties

The human brain is a complex network in multiple space and time scales with many important topology, which include small-world characteristics, modular and highly connected networks of the core brain areas (Sporns, 2011). Complex network usually contains regular, small-world and stochastic networks, and these networks are judged by cluster coefficient and characteristic path length. The rule network has higher cluster coefficient and longer characteristic path length, and the random network has shorter characteristic path length and lower cluster coefficient. However, the small-world lies between the rule and random networks, which not only has rule network similar to highly clustering coefficient, but also short feature path length similar to a random network (Liang et al., 2010). The small-world network is the best balance between global integration of brain function activities and global specialization, which supports the two most basic organizational principles of the human brain (functional integration and separation of functions) (Rubinov and Sporns, 2010). Small-world characteristics can keep the network efficient, specific modular information and fast global information transmission (He and Evans, 2010). Previous studies showed that small-world characteristics exist in brain structures and functional networks, and changes in topology properties may lead to a variety of neuropsychiatric disorders, such as Alzheimer’s disease (Supekar et al., 2008), depression (Zhang et al., 2011), epilepsy (Liao et al., 2010), and post-traumatic stress disorder (Lei et al., 2015). Although the topology properties of these diseases display various changes, as a general rule, the more the network topology properties deviate from small-world, the more disordered the brain function is.

Cluster coefficient and characteristic path length can all indicate that the process of transferring networks from small-world networks to rule network or random networks. Zhang found that the overall efficiency of the white matter network is reduced in T2DM, and characteristic path length is increased, and that the global properties and node efficiency of the central sulcus are positively correlated with executive function (Zhang et al., 2016); Reijmer found that global efficiency and cluster coefficient are reduced, and characteristic path length is increased. The abnormality of these structural networks is related to the slow speed of information processing observed in patients (Reijmer et al., 2013). These findings are essentially in agreement with the results observed in the diabetic brain network by Wang et al. (2016) by using the fMRI technique. In contrast to the results of the aforementioned study, in the present study we found that T2DM patients have higher normalized cluster coefficient and lower characteristic path length, which are typical features of a small-world network. Similar changes have been observed in MCI. For example, Wang et al. (2014) found there was a higher normalized cluster coefficient when sparsity threshold was in the range 0.10–0.18, but normalized characteristic path length was lower over the entire range of threshold. This means that the cognitive impairments of the diabetics that participated in the study were mild, and that they do not show the same small-world characteristics as healthy controls. The increasing of the cluster coefficient indicates that the local brain network is enhanced in patients with diabetes mellitus. However, the decrease of the characteristic pathway length indicates that the ability for information transfer between remote regions of the brain in patients with diabetes is enhanced. It is speculated that the brain function of patients with diabetes mellitus may be impaired in the early stages of cognitive impairment. Under compensatory mechanisms, its ability for local information and remote information processing is enhanced, showing the information processing mechanism consistent with MCI; but with the further development of diabetes (Wang et al., 2014). The impairment in brain function is more severe than in T2DM patients. Having lost the ability to compensate, its long-distance information transmission ability drops.

This study also found that patients with diabetes mellitus have higher global and local efficiency, which may mean that the functional integration ability of the whole brain network and the information processing ability of the local sub-networks are enhanced, which is in accordance with the small-world characteristics results. The changes of these network parameters were much ascribe to metabolic disturbance of diabetics, needing the whole brain network to compensation for the increase of the overall integration efficiency. However, inconsistent results were found in previous studies of diabetic brain network structure (Reijmer et al., 2013; Zhang et al., 2016), and this difference may stem from differences in subjects and modes. Although the trends of the changes in these network parameters are inconsistent, they all illustrate the abnormality of the brain network efficiency in patients with diabetes mellitus.

The changes in the global topological properties of these brain networks indicate the abnormality of neural network structure in diabetes mellitus. Given that the small-world characteristics are not affected, the brain network is thus compensated with the high efficiency by global integration and local separation after cognitive function of diabetics is impaired, which makes this characteristic more obvious than the normal person.

### Change of Node Properties

Consistent with the increased network efficiency, we also found the brain areas of the nodes in the patients with diabetes, the brain regions involved in the patients included the superior frontal gyrus, olfactory cortex, posterior cingulate gyrus, occipital gyrus and superior temporal gyrus. In functional studies of diabetes, the superior frontal gyrus was associated with cognitive impairment, and T2DM patients showed worse executive and memory skills in high working load memory tasks (Chen et al., 2014; Zhang et al., 2015). In addition, a reduction in the brain surface area of patients was also found in a previous study (Peng et al., 2015). Findings in the temporal gyrus and ITG in patients with diabetes mellitus are as follows: the cortex of cortical white matter decreased and was related to memory defects (Yau et al., 2009); the relationship between white matter damage and memory loss is closely related (Zhang J. et al., 2014); the decrease of functional activity is closely related to memory decline, and occurs earlier than atrophy (Zhou X. et al., 2014); these findings are consistent with the region of the advanced cognitive function. The node properties of the right temporal gyrus and ITG were positively correlated with BMI of T2DM patients, but obesity is a crucial factor affecting many metabolic disorders in patients with diabetes mellitus (Zhou and Xue, 2006). The results confirm that the changes of brain network topology in patients with diabetes have a relationship with obesity.

Previous studies have shown that the temporal and frontal lobes are the main brain areas involved in the cognitive impairment of diabetes. Evidence of structural and functional abnormalities of the frontal-temporal brain region in patients with diabetes mellitus is as follows: using diffusion tensor imaging (DTI) technology, Yau et al. (2009, 2014) found that the gray matter and white matter microscopic abnormalities of T2DM patients were mainly located in the frontal and temporal lobes, and participated in language memory disorders. Last et al. (2007) used continuous arterial spin label imaging to reveal temporal and frontal blood flow in T2DM patients. Using structural MRI and PET techniques, García-Casares observed a reduction in gray matter density and glucose metabolism in the fronto-temporal brain regions in T2DM patients after controlling for other vascular risk factors (Garcíacasares et al., 2014). Zhou et al. used rs-fMRI technology to detect diffuse amplitude of low frequency fluctuation (ALFF) changes in MCI patients with diabetes, including in the temporal lobe and frontal lobe (Zhou X. et al., 2014). These findings indicate that the fronto-temporal brain region is associated with impairments in cognitive functions such as information processing speed, memory and emotion (Yau et al., 2009, 2014; Hsu et al., 2012). The posterior cingulate gyrus, as the core node of the DMN, plays a key role in the cognitive process, and it is used to predict the biological markers of MCI converting to early Alzheimer’s disease. Recent studies have shown a reduction in the value of the posterior cingulate gyrus ALFF that is highly correlated to the MoCA scale, besides, its dysfunction of the functional connection with multiple brain is more indicative of the specific impairment of memory function (Qin et al., 2016). Studies conducted by Cui and others have also confirmed that the decrease of functional connectivity in the posterior cingulate cortex in the DMN is related to cognitive behavior (Cui et al., 2015). Therefore, it is speculated that the node properties abnormality in the posterior cingulate cortex is related to cognitive impairments in T2DM patients, such as information processing speed, emotion, memory and executive ability.

## Conclusion

This study examined the brain network topology of T2DM patients by using graph theory-based analysis of rs-fMRI, and found that both global and node properties were changed. Compared with healthy controls, the normalized cluster coefficient and characteristic path length showed stronger small-world characteristics, indicating that the impairment in cognitive function is slight, and multiple brain regions make up an efficient sub-network that compensates for the small-world characteristic of the normal human brain. The changes of node properties were most prominent in the temporal and frontal lobes, and posterior cingulate gyrus, and the abnormality of node properties in these brain areas is related to the changes of brain cognitive function, which is a similar finding to those in previous studies. Consequently, inferring the efficient integration of functional networks of T2DM patients, sub-networks composed of multiple brain regions compensate for their impairment of cognitive function. Thus, the global properties and node properties results are activated excessively. The main limitations of the present study include: (1) This study used the AAL template to divide the cerebrum into 90 brain regions (nodes), and omits the epencephal. In previous brain network research, there is no gold standard template for the division of brain regions. (70 brain regions of automatic matching and non-linear imaging anatomical marker mapping, 116 brain regions including epencephal, 90 brain regions adopted by this study). To avoid the influence of the varying node definitions of the different brain atlas templates, future studies should establish and use a unified standard template. (2) This study only examined the MRI data of diabetic patients in the resting state, and did not investigate the structural image data at the same time. The brain is a complex network system with both anatomical and functional connections. If a combination of brain function and structure of patients can be analyzed, more powerful evidence for the changes of the topological properties of the diabetic brain network will be obtained. Future studies should combine multi-modal imaging data to establish these relationships, and these will provide more accurate interpretation of the neural mechanisms of patients with diabetes mellitus. In short, the combination of fMRI and graph theory for investigation of brain function in diabetes has just begun. Future research will focus more on the complex function and connection network properties of the patient’s brain in the resting state, and will have potential significance for the early treatment, control and management of the disease and prevention of its complications. (3) Three diagnostic criteria for T2DM patients were published by WHO in 1999. One of them (OGTT) was used in this study, which may cause bias in the research results. In future studies, the other two criteria should be considered to improve the reliability of the results. (4) In this study, age and gender were used as covariates when performing correlation analysis between network properties and clinical parameters, but the diagnosis was not used as covariates. In future studies, clinical diagnostic parameters (such as BMI, etc.) should be used as covariates, which may improve the reliability of the results.

## Ethics Statement

This study was carried out in accordance with the recommendations of Sichuan University with written informed consent from all subjects. All subjects gave written informed consent in accordance with the Declaration of Helsinki. The study was also approved by the Medical Ethics Committee of Henan Provincial People’s Hospital.

## Author Contributions

JZ contributed to the conception of the study. JX and FC contributed significantly to analysis and manuscript preparation. JX, MW, and TL performed the data analyses and wrote the manuscript. JZ, TW, and HY contributed to the interpretation and discussion of the results of the analysis.

## Conflict of Interest Statement

The authors declare that the research was conducted in the absence of any commercial or financial relationships that could be construed as a potential conflict of interest.
